# The Integrative Life History of Maternal Effects

**DOI:** 10.1093/icb/icae117

**Published:** 2024-07-17

**Authors:** Jamie R Marks, Simon P Lailvaux

**Affiliations:** Department of Integrative Biology, Kellogg Biological Station, Michigan State University, 3700 E. Gull Lake Rd., Hickory Corners, MI 49060, USA; Department of Biological Sciences, University of New Orleans, New Orleans, LA 70148, USA

## Abstract

Context-dependent allocation of resources drives trade-offs among fitness-related traits and other phenotypes to which those traits are linked. In addition, the amount and type of acquired resources can also affect the phenotypes of other organisms through indirect genetic effects, as exemplified by the maternal provisioning of offspring. Despite a large literature on maternal effects, we lack a comprehensive understanding of the extent to which mothers might affect the phenotypes of their offspring, as well as the various mechanisms by which they do so, particularly with regard to many functional traits that are key determinants of survival and reproduction. Our goals in this paper are to review the various approaches to measuring and understanding maternal effects and to highlight some promising avenues for integration of maternal effects with some other key areas of evolutionary ecology. We focus especially on nutritional geometry; maternal age; and traits proximate to fitness such as whole-organism performance. Finally, we discuss the logistic and practical limits of quantifying these effects in many animal systems and emphasize the value of integrative approaches in understanding the mechanisms underlying maternal influence on offspring phenotypes.

## Introduction

Life history studies consider the scheduling of key fitness-enhancing tasks over individual lifetimes (Flatt and Heyland 2011). As such, life history theory deals with acquisition- and allocation-based trade-offs, whereby investment of limited acquired energetic resources into the expression of certain fitness-enhancing traits reallocates energy away from others (Stearns 1989). Life history theory is also an essential conceptual framework for studying maternal effects, a transgenerational phenomenon whereby a mother’s environment and or/underlying genotype influences the phenotype of her offspring (Mousseau and Fox 1998; Wolf and Wade 2009). Wolf and Wade (2009) explicitly define maternal effects as the causal influence of the maternal genotype or phenotype on the offspring phenotype. This definition acknowledges and encompasses maternal effects that are driven by both direct and indirect genetic effects (McGlothlin and Brodie 2009; McAdam et al. 2014; see also Keaney et al. 2020), and by plastic responses on the part of the offspring to variation in the maternal physiological state. In both cases, life history plays an important role in both effecting and facilitating such responses.

The origins and maintenance of phenotypic variation are of interest to evolutionary biologists because it is the phenotype that is the primary target of selection (Arnold 1983; reviewed in Svensson 2023). Although the relative effects of additive genetic and environmental variation on phenotypic trait expression have historically received a great deal of attention, there has been a growing recognition of the potentially important contribution of various mechanisms of nongenetic inheritance to such expression as well (Bonduriansky and Day 2018). Of these mechanisms, the influence of maternal identity on the offspring phenotype is an important source of phenotypic variation. Thus, the dynamic maternal state, which is itself influenced by current environmental conditions, is linked to the multiple aspects of the overall offspring phenotype, any of which might covary or trade-off against each other via several potential mechanistic pathways (Ghalambor et al. 2004; Garland et al. 2022; Husak 2022). Despite the ubiquity of these maternal effects, the known scope of maternal provisioning to and inheritance of offspring phenotypes remains limited to those traits that, while generally easily measured, are not necessarily the most proximate to fitness. Furthermore, the identity and function of those physiological pathways underlying the maternal influence on these key offspring phenotypes have historically been obscure.

Our goal in this paper is not to comprehensively review the large maternal effects literature, but to briefly highlight some important approaches to understanding the processes and mechanisms linking variation in the maternal phenotype to variation in offspring phenotypes from an explicitly integrative, life history perspective. In addition, we also suggest some promising future avenues to study maternal effects from perspectives that have historically received little attention. We focus specifically on those topics that are, in our opinion, understudied in this context as opposed to those that are dealt with extensively elsewhere, such as mitochondrial function (Pick et al. 2016; Kim et al. 2022).

### Measuring maternal effects

Maternal effects are ubiquitous and can have both short- and long-term effects on offspring phenotypes. In a recent survey, Moore et al. (2019) found that over 10% of phenotypic variation is due to maternal effects across a range of organisms and taxa. Although strongest on traits expressed early in an individual’s lifetime, such effects can also persist into adulthood. Estimating maternal effects should therefore be a priority, and researchers have employed several strategies to do so. The quantitative genetic approach partitions phenotypic variation into genetic and environmental variation, which can comprise maternal genetic (i.e., the effect of genes expressed by the mother on the offspring phenotype) and maternal environmental effects (i.e., influence of the environment provided by the mother on offspring phenotypes), respectively (Wilson et al. 2010; Roff 2012). Early interest in maternal effects stemmed from a desire to control for them as a potential source of bias in estimates of heritability and additive genetic variation, but evolutionary biologists have subsequently recognized their genetic basis and thus their potential for altering evolutionary trajectories (McAdam et al. 2014).

Maternal effects can be estimated from either a breeding design or from an appropriate pedigree (see Wilson et al. 2010 for an overview of pedigree analysis using the animal model), as can the correlations among suites of traits, which again have both maternal and maternal genetic components. However, both the maternal environmental and maternal genetic variance components cannot always be estimated from opportunistic or reconstructed pedigrees, which can be limited either by sampling and/or by the mating system of the animal, or both. Similarly, many species are not amenable to manipulative breeding designs. An alternative approach to measuring maternal effects is to manipulate some aspect of maternal physiology to elicit trade-offs in the maternal phenotype and then to examine the offspring phenotype. Although this approach confounds additive genetic, maternal environmental, and maternal genetic effects, it is still useful for understanding the consequences of maternal variation on the offspring phenotype, particularly in species that are not amenable to breeding manipulation. An ideal approach would be to combine both methods into a genotype-by-environment breeding experiment, which involves conducting multiple breeding designs under different levels of environmental variation. This is difficult in practice and is rarely done. Recent studies have made increasing use of genomic data to estimate “classic” quantitative genetic parameters from whole genomes (Johnston et al. 2022). These approaches hold a great deal of promise for understanding maternal and maternal genetic variation of select phenotypes.

### Dietary restriction and nutritional geometry

The quintessential life history trade-off is that between reproduction and survival, which diet restriction can promote (Weindruch and Sohal 1997; Fontana et al. 2010; Marks et al. 2023). Defined as undernutrition without malnutrition, dietary or caloric restriction is an ubiquitous conserved mechanism that catalyzes life history trade-offs in animals ranging from nematodes to vertebrates (reviewed in Mair and Dillon 2008). As such, it is one of the most commonly used research methods to manipulate the maternal energetic environment and promote maternal effects. If a mother has limited food, this can lead to changes in offspring phenotype such as smaller clutch sizes in some birds (Hořák et al. 2015) and insects (Jann and Ward 1999), or larger, but fewer, offspring in lizards such as green anoles (*Anolis carolinensis*) (Marks et al. 2023). Complicating our understanding of the consequences of resource availability for maternal effects is the concept of nutritional geometry.

The nutritional geometry diet framework conceptualizes animal diets in terms of a multivariate nutrient space, whereby each axis or plane within that space represents a distinct functionally relevant nutrient (Simpson and Raubenheimer 1993; Raubenheimer and Simpson 1999). The ratio of macronutrients (i.e., fats, proteins, and carbohydrates) consumed is particularly important to this framework, and studies have documented species-, sex-, and age-specific macronutrient ratio intake targets with empirical consequences for life history (Jang and Lee 2018), reproduction (South et al. 2011), aging (Zajitschek et al. 2012), and survival (Moatt et al. 2019). The sex-specificity is particularly of note, as adult males and females frequently exhibit different intake targets to optimize reproductive effort and likely residual reproductive value (RRV) as well (Maklakov et al. 2008, 2009; Rapkin et al. 2018). In black field crickets (*Teleogryllus commodus*), for example, male and female reproductive effort is optimized on different diets, with males both preferring and exhibiting higher reproductive effort on a carbohydrate-rich diet, whereas female reproductive investment is maximized on a high protein diet (Maklakov et al. 2008, 2009). Rapkin et al. (2018) later found that dietary macronutrient ratio mediates the trade-off between reproductive effort and immune function in a sex-specific fashion in the decorated cricket, *Gryllodes sigillatus*.

Despite these documented sex-specific effects of macronutrient intake on both key fitness-related phenotypes and the relationships among them, relatively few studies have considered the effects of maternal variation on offspring phenotypes from an explicitly nutritional geometry perspective. Bonduriansky et al. (2016) found that maternal protein consumption positively affected egg hatching success and offspring growth in *Teleostylinus angusticollis* flies, whereas paternal effects were driven by male carbohydrate intake. Similarly, Mair et al. (2005) found that a decrease in maternal protein consumption specifically extended lifespan in flies (see also Strilbytska et al. 2020). Beyond the general effect of protein, recent evidence implicates specific amino acids in driving the general trade-off between longevity and reproduction via their effects on mTOR (mammalian target of rapamycin)—a highly conserved central pathway for cell growth and metabolism in eukaryotes (Hall 2008). In progeroid mice that have been genetically modified to exhibit a phenotype of an aged animal beyond their actual age, where this mechanism is commonly studied, three specific branched-chain amino acids (leucine, isoleucine, and valine) must be reduced to increase median lifespan (Richardson et al. 2021). Given the widespread relevance of nutritional geometry to life history, researchers who are interested in properly understanding the impact of maternal diet on offspring phenotype, might consider manipulating macronutrient ratios, and potentially branched-chain amino acid availability, to understand how this variation in the maternal nutritional regime affects not only her own life history, but that of her offspring as well. These proposed studies are crucial to filling in gaps in our knowledge related to these concepts.

### Mechanisms, trade-offs, and stress

The efficacy of dietary restriction and macronutrient variation to induce differential investment in survival vs reproduction, and thus to mediate life-histories, is linked primarily to the insulin/insulin-like signaling network (IIS) (Dantzer and Swanson 2012). This highly conserved pathway is crucial for cell growth and metabolism via the action of two primary hormones, namely insulin-like growth factor 1 (IGF1) and insulin-like growth factor 2 (IGF2). IGF1 is positively correlated with growth and receives much attention because of its suspected anti-aging and potential anti-cancer properties (Swanson and Dantzer 2014; Ianza et al. 2021). By contrast, IGF2 is not as well characterized. IGF2 is typically referenced only in the context of embryonic development because some lab rodents, where it is heavily studied, do not exhibit IGF2 postnatally (Schwartz and Bronikowski 2016; Beatty and Schwartz 2020).

Although typically conceived as a nutrient-sensing pathway (Barbieri et al. 2003), Regan et al. (2020) emphasize that the IIS is sensitive to a range of environment variables and show that different external pressures can trigger distinct responses. Indeed, variation in the maternal environment other than that induced by diet can impact maternal *IGF* expression (Regan et al. 2020). For instance, the IIS is also sensitive to water availability via the *Pickpocket28* gene (Waterson et al. 2014). Furthermore, there is mounting evidence that *IGF2* is not only expressed postnatally in non-murine animals (Beatty et al. 2022), but is also sensitive to environmental variation, potentially more so than *IGF1*. For example, hepatic expression of *IGF1* and *IGF2* of adult green anole females was significantly different between those that were exposed to food limitation (low diet animals received 1 cricket 3 times/week as opposed to high diet animals who received 3 crickets 3 times/week) for 8 weeks (Marks et al. 2021) and those that were trained for sprinting ability for 6 weeks (Marks et al. 2022). Marks (2023) later found that the offspring of sprint-trained mothers exhibited longer snout-vent lengths than those from the untrained moms. Although the underlying mechanism driving this result is not known, there is evidence that IGF transcripts can be allocated to offspring in other species such as haplochromine fish (Ahi et al. 2018). There is also evidence of other transcripts beyond IGF being allocated to eggs, such as transcripts for growth hormone receptors and the glucocorticoid (GC) receptor (Ahi et al. 2018). Indeed, mothers can allocate transcripts implicated in physiological processes such as growth, metabolism, and reproduction; consequently, future experiments should test if allocation of transcripts could change across different environmental conditions or when maternal functional traits are manipulated.

A further potentially important pathway that impacts maternal variation is the hypothalamic–pituitary–adrenal (HPA) axis. The primary function of the HPA axis is to maintain physiological homeostasis in response to various external and internal stimuli (Stephens and Wand 2012). GCs (cortisol in most mammals and corticosterone in rodents, birds, and other reptiles) (Botía et al. 2023) are the principally produced hormones of this network, and these are typically referenced as the hormones implicated in a stress response (Cockrem 2007; MacDougall-Shackleton et al. 2019; Sadoul and Geffroy 2019). Variation in maternal circulating hormones, such as those pertaining to the HPA, is relevant to maternal effects because there is evidence of maternal hormone allocation to offspring. Although there is a large literature on hormone-mediated maternal effects, including in mammals (Maestripieri and Mateo 2009; Edwards et al. 2021), we highlight hormone-mediated maternal effects in oviparous animals here, particularly within the context of diet manipulations. Maternal steroids can be deposited into yolk via vitellogenesis or during yolk protein formation (Moore and Johnston 2008). They can also be deposited when the egg is in the oviduct (Johnston and Moore 2005; Moore and Johnston 2008). Because diet restriction is frequently used as a stressor, maternal circulating GC levels might be expected to increase under this scenario, along with a potential concomitant increase in allocation of this hormone to increase to their offspring (although it is also important to note that yolk steroid levels at one time point are not representative of the mother’s steroid concentrations in plasma at that same time since GC deposition by mothers is unidirectional [Moore and Johnston 2008]). For instance, Hanover et al. (2019) showed that when green anole females were diet restricted, their circulating levels were not statistically different from those fed a normal diet, but corticosterone levels decreased in eggs. Rather than releasing more corticosterone in response to this poor environment, diet restriction may have promoted a trade-off whereby retention of GCs was necessary to maintain normal functional traits. Alternatively, it is possible that the embryos themselves were metabolizing corticosterone differently. Importantly, these hormones are implicated in numerous physiological processes (Love et al. 2004), and a lack in their response to an environment seen as challenging (stressful) could also mean that other mechanisms that promote variation in offspring phenotype are implicated beyond the HPA axis to promote variation in offspring phenotype.

Several of the above mechanisms discussed, especially corticosterone and the HPA axis, are frequently implicated in the maternal response to stress. However, the concept of “stress” is ill-defined, and considerable controversy exists in the literature over the meaning of the term (see Borics et al. 2013; MacDougall-Shackleton et al. 2019). One potentially fruitful approach is therefore to target specific pathways or physiological mechanisms that are likely to be involved in maternal manipulations or that may underlie known resulting trade-offs (reviewed in Husak 2022). Doing so is particularly important when employing novel manipulations or for interpreting the results of experiments that could conceivably promote unintended maternal effects.

Finally, it is important to note that because maternal effects are a transgenerational phenomenon, they fall under the umbrella of epigenetic effects. Epigenetic effects are those in which non-genomic material is transferred from parent to offspring (Ho 2014) (not to be confused with epigenetic inheritance, where these changes are heritable for one or more generations [Lind and Spagopoulou 2018]). Although the specific epigenetic mechanisms remain to be elucidated, Ho and Burggren (2012) demonstrated that hypoxia-exposed zebrafish females have significantly lower egg volume than those not exposed to these conditions and it is likely that underlying changes in gene expression are responsible for this phenotypic effect on offspring. They also emphasize that there is evidence of similar epigenetic modifications in vertebrates yet more work is needed to understand when and how epigenetic effects occur (Ho and Burggren 2012).

### Age-dependent maternal effects

A further layer of complexity potentially influencing these mechanisms and pathways is maternal age. Because life history trade-offs are dynamic decisions that organisms make throughout their lifetime, age can strongly influence how an animal allocates resources to offspring. Although the reproductive consequences of male age have been of interest from the perspective of sexual selection in general, and female choice in particular (Brooks and Kemp 2001), maternal age has long been recognized as a driver of maternal effects (Priest et al. 2002). For example, a decrease in offspring survival as a function of increasing maternal age is known as the Lansing effect, and similar negative effects on offspring health and lifespan have been demonstrated in a variety of taxa ranging from rotifers (Jennings and Lynch 1928; Lansing 1954) to mammals (but see Ivimey-Cook et al. 2023, who show that taxonomic biases in published studies may skew this perspective). Despite this interest, the mechanisms of these age effects remain poorly understood. Moreover, comparative studies examining effects of maternal age can further be complicated by how age is defined within a specific species (Riddle et al. 2023). For example, a chicken’s (*Gallus gallas*) age is based on the time since hatching (McCartney 1978), whereas zebrafish (*Danio rerio*) age is characterized as the time since fertilization (Singleman and Holtzman 2014; Riddle et al. 2023). Other allocation decisions may be largely independent of age, driven instead by a change in the extrinsic mortality risk. For example, terminal investment describes increased allocation to reproduction when survival prospects diminish (Duffield et al. 2017), as opposed to the classic senescent pattern whereby such investment decreases with advancing age.

Understanding age-dependent changes in the manner and degree in which maternal effects impact offspring phenotype is also a central pillar in obstetrics. In humans, we know that maternal age plays a large role in fertility and fetal health (Van Noord-Zaadstra et al. 1991; García et al. 2018; Delbaere et al. 2020). For example, there is a higher likelihood of preterm birth with advanced maternal age (Esposito et al. 2022). Unfortunately, age-dependent risks are rarely labeled correctly as maternal effects, but doing so could be an important step toward bettering our understanding of maternal effects as a whole, especially in long-lived animals such as humans. Similar to humans, older female painted turtles (*Chrysemys picta*) also exhibit a decline in reproductive abilities (Warner et al. 2016), yet hatchling survival increases with maternal age (Paitz et al. 2007). In other animals such as mites (*Sancassania berlesei*), fecundity drops with age, but regardless of the resource environment, older females lay heavier eggs compared to the younger mites (Plaistow et al. 2007). Plaistow et al. proposed that this maternal effect could potentially be adaptive if the larger egg size helps with competition among offspring from younger females, but a comprehensive breeding design would be needed to appropriately test this (Plaistow et al. 2007). Finally, a large literature shows that female allocation of resources is age-dependent and can be altered by factors such as diet or mating frequency (e.g., Lailvaux et al. 2011). Collectively, these studies suggest that maternal age is likely to be an important modifier of maternal effects and should be explicitly considered in relevant studies where feasible.

### Functional traits

While studies of maternal effects are common, the types of traits that have been measured in such studies tend to be biased toward a specific subset of phenotypes, especially morphology. Traits related to animal function, however, such as whole-organism performance traits, are seldom considered. Whole-organism performance traits are measures of animal function defined as quantitative measures of an animal conducting a dynamic, ecologically relevant task such as running, jumping, biting, or flying (Bennett and Huey 1990; Lailvaux and Irschick 2006; Irschick et al. 2008). Moore et al. (2019) synthesized 151 studies considering the strength of maternal effects in animal populations and found that only three such studies meeting their criteria for inclusion were conducted on performance traits as defined above, with morphology and even behavior tending to be much more commonly measured. This distinction is important because Arnold's  (1983) influential ecomorphological paradigm states that performance traits are more proximate to fitness, and thus direct targets of selection, compared to traits at lower levels of biological organization, such as morphology. Changes in morphology therefore tend to follow functional requirements, although performance itself may be further modulated by behavior before affecting fitness (Garland and Losos 1994; Orr and Garland 2017). Performance measurement can be time consuming and often requires specialized equipment (Losos et al. 2002); nonetheless, measuring performance in addition to morphology is important for several reasons.

Although the performance gradient is easily conceptualized as a univariate relationship between a performance trait and its underlying morphology, animals are frequently required to conduct different kinds of performance (related to tasks such as foraging, mating, predator evasion, etc.), which can place conflicting demands on the underlying morphology and physiology (Van Damme and Wilson 2002; Pasi and Carrier 2003). The performance gradient is thus inherently multivariate because a given morphological trait supports multiple different performance capacities, which can drive trade-offs among performance traits (Bergmann and McElroy 2014; Lailvaux et al. 2022; Bergmann and Tonelli-Sippel 2024). The situation is further complicated in species exhibiting marked sexual dimorphism, where multivariate relationships between morphology and performance and their resulting functional trade-offs can be sex-specific (Simon et al. 2022). In addition to functional trade-offs among multiple performance traits, an emerging literature shows that any given performance trait might also trade-off with other phenotypes besides performance (Ghalambor et al. 2004; reviewed in Lailvaux 2014). For example, training green anole lizards for different types of locomotor performance (i.e., sprinting and endurance) induces trade-offs with traits such as immune function or growth (Husak et al. 2016, 2017) that are both sex-specific and specific to the type of performance trained, and activation of immune activity impinges upon locomotor performance as well (Husak et al. 2021). Maternal investment can therefore have potential consequences for offspring phenotypes at several multiple levels of biological organization beyond simple morphology, including other functional and phenotypic traits that rely on either a common acquired resource pool or shared mechanistic pathways (Lailvaux and Husak 2014; Husak and Lailvaux 2022).

Despite the relevance of multivariate performance to fitness, the multivariate performance gradient is seldom measured (Lailvaux 2022), particularly within the context of maternal effects. Consequently, few studies have rigorously considered the impact of maternal identity on offspring performance. Zablocki-Thomas et al. (2021) used an animal model approach to estimate the genetic relationships between head morphology and bite force and between limb morphology and pulling force in the gray mouse lemur (*Microcebus murinus*). Significant maternal effects were found for multiple aspects of the respective performance gradients; but although Zablocki-Thomas et al. (2021) found a significant genetic correlation between head morphology and bite force, the maternal correlation between these same traits was not significant. Furthermore, the study was based on a captive population, with specific housing conditions, which meant there was no opportunity for females to mate with multiple males (Zablocki-Thomas et al. 2021). Consequently, the additive maternal genetic effect (i.e., the effect of the maternal genotype on an individual’s phenotype independent of the direct effect of inherited genes; see Wilson and Reale 2006) could not be estimated. Despite these caveats, this study shows that functional traits, as well as the performance gradient, can feasibly be investigated from the perspective of maternal effects, although such traits are known to vary both in their heritabilities and in the significance of genetic correlations among traits (Lailvaux 2010; but see Ginot et al. 2022). The contribution of maternal identity to multivariate suites of traits therefore remains unclear at present. However, understanding such contributions is likely to be important if we are to fully understand the evolutionary trajectories of whole-organism performance traits in particular.

### Limitations and future avenues

Life history provides an appropriate conceptual framework for understanding maternal effects, but such studies are nonetheless subject to limitations. Many mechanisms facilitating maternal effects are still unresolved, as are those mechanisms underlying key life history trade-offs (Husak and Lailvaux 2022). Therefore, it is crucial to think broadly about how different underlying mechanisms may be affected by the environmental pressures experienced by a female. To give an example, from a functional perspective, if the effects of activity or exercise on some component of offspring phenotype are being tested, it is necessary to consider the appropriate underlying physiological processes. This is because aerobic performance capacities such as endurance are bolstered by different physiological pathways and mechanisms than anaerobic capacities such as sprinting, and these physiological differences could consequently drive distinct sets of trade-offs as well, depending on the type of performance in question. Even if specific mechanistic (or molecular) pathways cannot be easily assessed, the choice of manipulations applied and phenotypes measured can provide important insights into the putative underlying pathways underlying specific maternal effects. The application of breeding designs or pedigrees, although logistically challenging, nonetheless holds enormous opportunity for rigorously estimating the influence of the maternal genotype and phenotype on offspring traits. Despite this, their application is all too frequently limited to select phenotypes such as morphology that, although more easily measured than others, arguably reveal little regarding the specific mechanisms linking maternal and offspring variation. Similarly, studies of nutritional geometry and rigorous estimation of the macronutrient space may be demanding to execute for many vertebrate species, but nonetheless are promising avenues for illuminating the role of mechanistic pathways such as the IIS in affecting variation in offspring phenotypes and life-histories.

Although key to life-histories in general, one further component to maternal effects that is seldom considered, albeit necessary to understand the ecological and evolutionary consequences of resource allocation, is the concept of RRV (residual reproductive value) (Williams 1966). Defined as the expected reproductive capacity remaining in an animal’s life, RRV can impact proximate decisions regarding investment in reproduction versus survival, as well as any accompanying life history trade-offs in other aspects of the integrative organismal phenotype. RRV is typically studied at the population level (Delaney et al. 2021); however, because a mother’s investment in herself and her offspring is dynamic and can change in concert with age and environmental factors, one fruitful approach might be to consider RRV at the individual level, as well. Such an approach will also provide a better understanding of how these factors can impact maternal effects and ultimately maternal fitness.

Finally, a number of molecular/hormonal integrator pathways could potentially facilitate maternal effects but have received scant attention from the perspective of maternal investment. For example, the roles of molecules such as myokines in facilitating performance-related allocation trade-offs tend to be little-studied beyond a handful of model systems (reviewed in Husak and Lailvaux 2022). Consequently, our understanding of whether and how they might mediate maternal effects is also lacking (Goudreau et al. 2021).

Despite these obstacles, there is an enormous opportunity to increase our understanding of the various ways in which maternal identity affects offspring fitness and phenotypes. Integrative approaches that combine data on organism function, physiological mechanisms, and different levels and methods for manipulating various types of environmental variation are also opportunities for collaboration. These approaches, compounded with wider recognition and acknowledgement of maternal effects across diverse disciplines of science, will help bridge these gaps and foster a more comprehensive understanding of this ubiquitous phenomenon.

## Notes

From the symposium “What do trade-offs mean to reproducing females?: An integrative look at whole-organism trade-offs” presented at the annual meeting of the Society for Integrative and Comparative Biology, January 3 to January 7, 2024.
